# Bibliometric study on artistic swimming

**DOI:** 10.3389/fspor.2023.1196144

**Published:** 2023-09-15

**Authors:** Ane Begoñe Rincón, Alfonso Trinidad, Alejandro López-Valenciano

**Affiliations:** ^1^ Aqualab Research Group, Departamento de Educación y de Humanidades, Facultad de Ciencias Sociales y de Comunicación, Universidad Europea de Madrid, Madrid, Spain; ^2^ Department of Education Science, Universidad Cardenal Herrera-CEU, CEU Universities, Castellon de la Plana, Spain

**Keywords:** bibliometrics, artistic swimming, injuries, sport, research

## Abstract

**Introduction:**

The objective of this paper is to study the research trends in the sport modality of artistic swimming and to analyse the scientific production regarding this sport.

**Methods:**

Two hundred and twenty articles related to the theme were selected after a search in the PubMed, SPORTDiscus and Web of Science databases and some additional records, using the theme “synchronised swimming OR artistic swimming” up to December 2022. The variables scrutinized were the following: (1) title, (2) discipline, (3) type, (4) sample size and (5) sporting level.

**Results:**

The results indicate that (1) 53.6% of the artistic swimming articles were case studies, (2) 23.2% talked about physiology, (3) 32.7% had a sample of 11–50 participants, and (4) 30.9% of the sample was elite/international swimmers.

**Discussion:**

Over the years there has been a considerable increase in studies on artistic swimming; the topics of greatest interest in artistic swimming have been physiology, sports performance and injuries. Even so, it seems that for the moment artistic swimming has little impact, probably due to its status as a minority sport and it having limited social and economic impact.

## Introduction

Physical activity and sports sciences have experienced a huge rise in recent decades, becoming one of the scientific branches with the highest number of publications per year. This has made it possible to carry out reviews and meta-analyses that compile the greatest findings in each discipline and thus improve different areas of sports training and injury prevention in most sports disciplines, such as football, basketball or tennis (1, 2). However, other sports considered minority ones have not had this level of repercussion and therefore there is no documentation that describes all the scientific literature and help available to professionals in a sports area as occurs in the sport of artistic swimming.

Artistic swimming is an Olympic sport that involves swimming, dance and ballet. Swimmers (solo, duet or team) perform a synchronised routine of elaborate movements in and under the water, accompanied by music (3). Its practice requires demanding advanced skills, such as great strength, stamina, flexibility, grace, artistry and precise synchronisation (3, 4), but given the multidisciplinary nature of this sport (5), it is necessary to know exactly the fields in which scientific production develops.

To create a systematic review or meta-analysis on artistic swimming that allows us to synthesise the results of different empirical studies on the effect of one or more variables on a precise final result, the existence of a significant and representative number of investigations on the subject is necessary. As it is a minority sport with many aspects to work on but with few resources in its facilities (3), artistic swimming does not arouse great scientific appeal and does not allow for revisions with this level of importance. Despite this, it is an increasingly well-known sport with a notable growth in licences in some countries, which has allowed an increase in research in recent years that needs to be synthesised and compiled. In addition, other tools allow the analysis of existing research on artistic swimming, such as bibliometric studies. Knowing and/or being able to quantify the scientific production of a certain area arouses great interest in the research community, which is why bibliometric techniques enjoy great applicability by theorists and/or scholars of any branch of scientific knowledge (6), in this way, experts in the area consider bibliometric studies on artistic swimming as the future result of a scientific development that will gradually adapt to the needs of the sport.

The creation of said documents or scientific studies, their analysis and the criticism carried out by the community itself are essential to building knowledge about the research activity, thus making the examiner's work credible (7). The analysis shows where the researchers are focused, making clear that the drawbacks are the main lines of action and research trends (6–9). Given the absence of reviews or bibliometric studies that compile and describe the existing literature on artistic swimming, the main objective of this research is to perform a bibliometric analysis of artistic swimming that allows knowing the research trends and analysing the scientific production regarding this sport.

## Methods

### Design and procedures

The bibliographic search was carried out in the PubMed, Web of Science, and SPORTDiscus databases. Subsequently, a search for additional resources was carried out through Google Scholar or based on the articles from primary sources. The following keywords in English “synchronised swimming” and “artistic swimming” and “natación sincronizada” and “natación artística” in Spanish were used and were combined using AND/OR Boolean markers up to December 2022.

As inclusion criteria, all articles that included a sample of synchronised swimming or artistic swimming athletes in any branch of the sport in scientific documents were selected. For the storage and organisation of the selected studies, a database was generated that can be obtained from the corresponding author.

### Data collection and analysis

**Figure 1:**
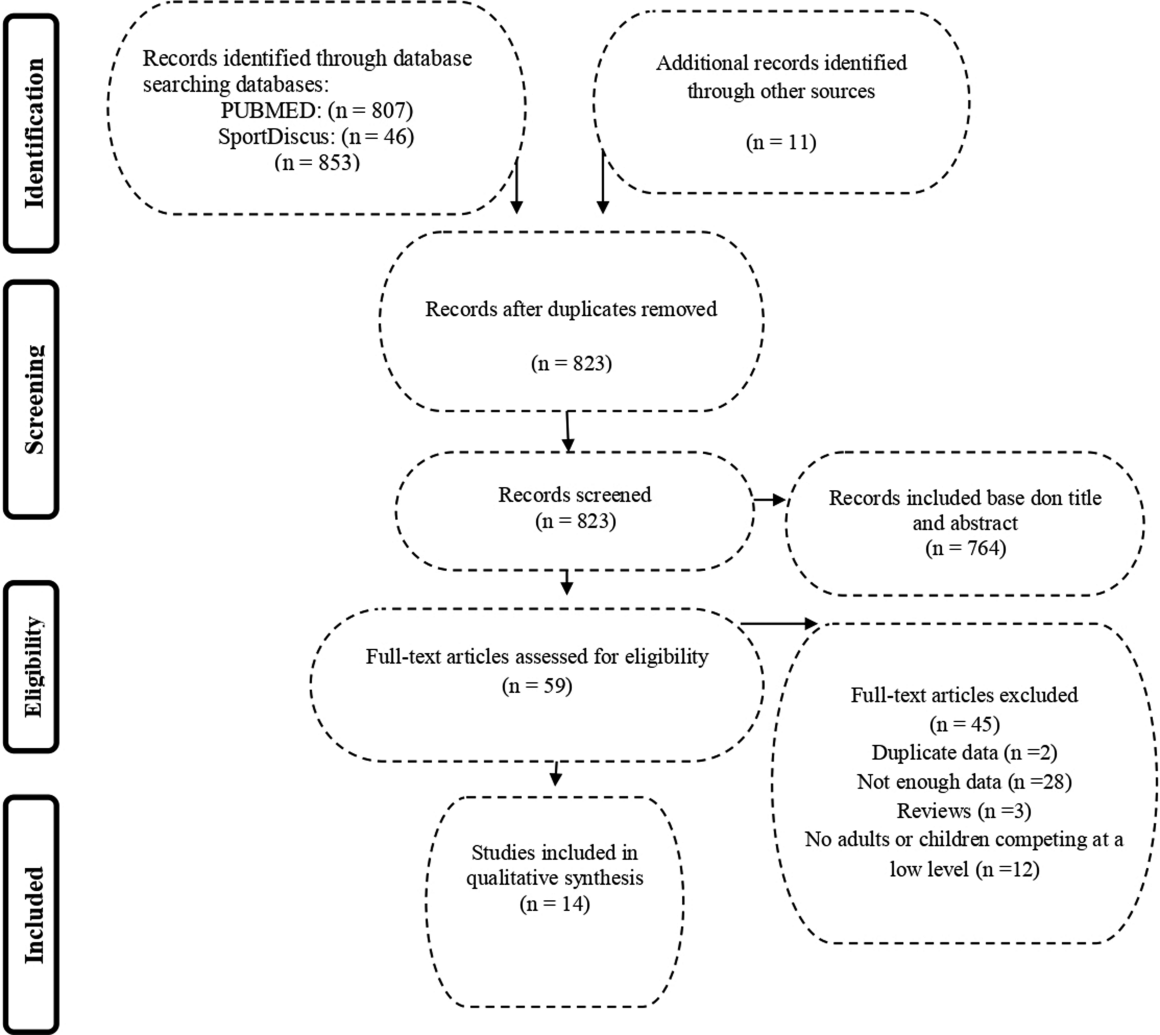
Flow chart of the selection of studies in the system review.

The initial search returned 537 results identified in the PubMed database, 1,837 results found in the SPORTDiscus database and 574 recognised results in the Web of Science database. Three hundred and twenty-eight articles from the different platforms were selected for a detailed evaluation of them (*n* = 328). One hundred and eight of the initial 328 studies were duplicates; therefore, the final study sample was 220 publications: 107 from PubMed, 81 from SPORTDiscus, 16 from Web of Science and 16 from additional sources. The flow diagram of the selection of studies for the review is shown in Figure 1.

The following variables were recorded and analysed concerning 5 categories and 40 different subcategories within the theme: (1) title, (2) main discipline of the study (injuries, sports performance, physiology, physical analysis, physical analysis for talent detection, body dissatisfaction and eating disorders, historical data, artistic swimming technique analysis, training theory, physical activity and health, biomechanics, doping and other substances, psychology, nutrition, didactics and/or others), (3) type of study (correlational, case, experimental, documentary, longitudinal and/or descriptive), (4) sample size and (5) sporting level of the sample (initiation, regional, national, elite/international or several). The classification of the categories associated with these variables was carried out based on the category systems used by various authors (10–13).

To assess the inter-reviewer reliability of the coding process, two researchers performed the search and coding process for the articles. For qualitative variables, Cohen's kappa coefficients were applied. On average, the Kappa coefficient was 0.86 (range: 0.81–0.97), which can be considered highly satisfactory. Inconsistencies between the two coders were resolved by consensus, and when the inconsistency was due to database ambiguity, this was corrected. Any disagreements were resolved by mutual consent in consultation with a third review author.

## Results

**Figure 2:**
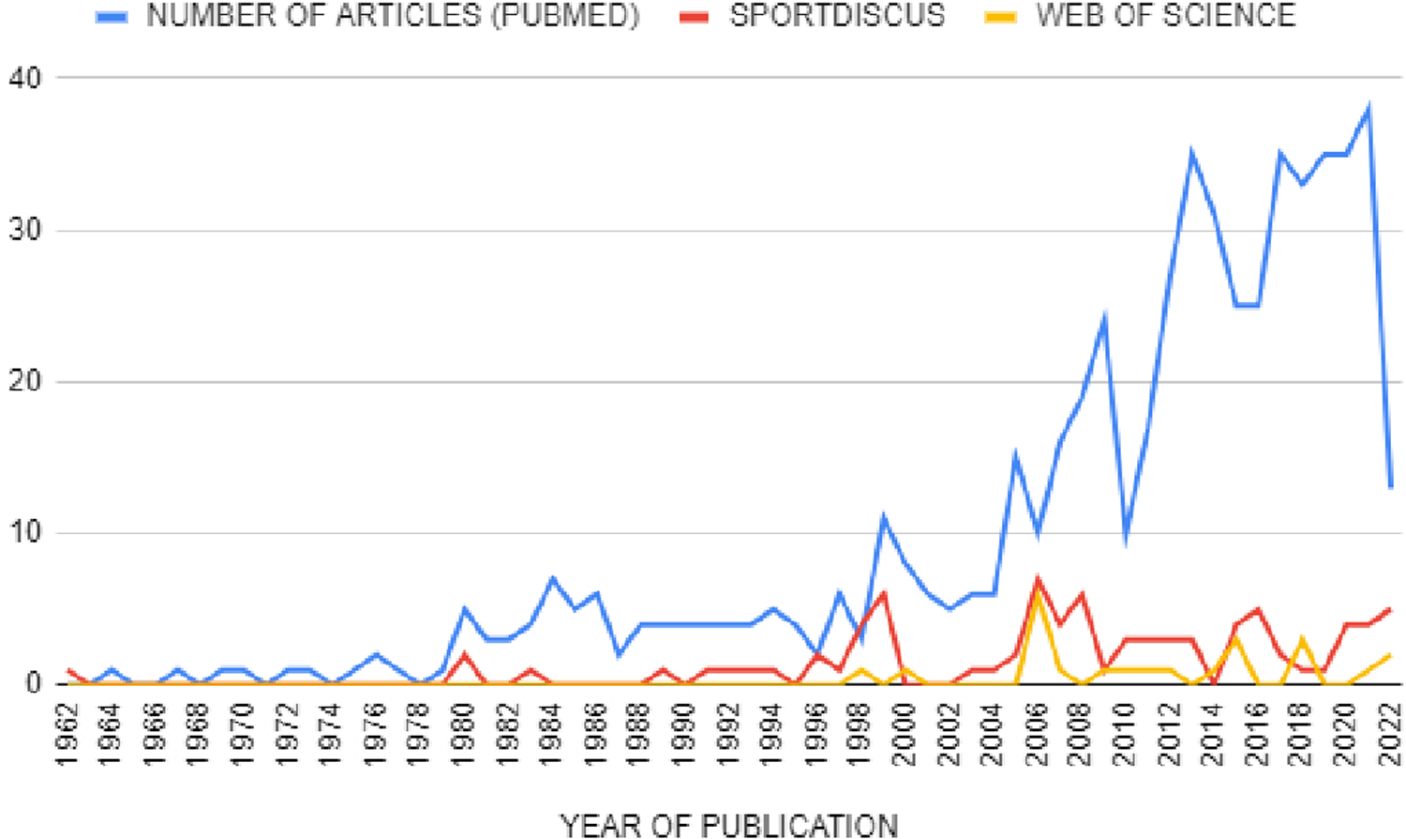
Evolution of the number of publications in PubMed, SportDiscus and Web of Science in artistic swimming.

As Figure 2 shows, three periods are clearly differentiated in terms of the evolution of the number of publications. In the first period, from 1962 to 1979, the number of publications on artistic swimming is very low. In the second period, from 1980 to 2004 approximately, there is a slight increase in the number of publications (2–11 publications per year). And finally, in the third period from 2005 to 2022, the level of production increases in a very notable way, reaching a maximum level of publications in 2021 (with a total of 43 publications). Regarding the language of publication, 210 of the 220 papers were published in English and 10 in Spanish. Of all the information presented, no study included male subjects.

The results shown in Table 1 show that there is a high percentage of case studies in artistic swimming in the last almost 60 years, whose purpose is to analyse an aspect of the subject in a single athlete or a very small sample of them (53.6%). Also worth noting is the importance of documentary research (selected publications on a specific topic that analyse the information and present it in a discussion) and conclusions, with the second highest percentage (16.8%).

**Table 1:** Type of publications.

Discipline	Count	%
Case study	118	53.6
Documentary research	37	16.8
Correlational study	26	11.8
Experimental study	19	8.6
Descriptive study	14	6.4
Longitudinal study	12	5.5

Studies on physiology are the main theme of the studies conducted (23.1%), followed by sports performance (12.2%). A wide range of other disciplines also provide information on different topics but with lower percentages, such as injuries, analysis of artistic swimming technique, physical analysis and/or historical data (Table 2).

**Table 2:** Main disciplines of the studies analysed.

Discipline	Count	%
Physiology	51	23.2
Sports performance	27	12.3
Injuries	26	11.8
Analysis of the technique in artistic swimming	18	8.2
Physical analysis^a^	15	6.8
Historical facts	14	6.4
Physical activity and health	12	5.5
Others^b^	12	5.5
Physical analysis for talent detection	10	4.5
Biomechanics	10	4.5
Body dissatisfaction and eating disorders^b^	10	4.5
Psychology	6	2.7
Training theory	5	2.3
Nutrition	4	1.8
Doping and other substances	3	1.4
Didactics	2	0.9

^a^ Analysis of different physical capacities, such as speed, coordination, explosive force, elastic force, reactive force, resistance or analysis of the physique of artistic swimming swimmers.

^b^ This category includes papers such as sports judging, score review in competition… which do not have enough articles to create a new category.

The number of swimmers analysed in each study varies remarkably (Table 3), with a high percentage of studies that had a sample of 11–50 participants (32.7%). Likewise, it is worth noting the studies that do analyse subjects but do not detail how many people the group is made up of (20% of the total articles collected). There are few investigations with large samples since only 7.2% correspond to studies with 51–100 swimmers or more than 100. As an exceptional fact, it should be noted that the study with the largest sample is the one that was conducted by Prien et al. (14) in 2017 with 1,194 participants, which analyses and compares the results of three FINA World Championships to see the risk of injury in the competition of this sport.

**Table 3:** Sample size of the studies analysed in artistic swimming.

Sample intervals	Count	%
No sample^a^	39	17.7
Doesn't specify sample size^b^	44	20
From 1 to 10	32	14.5
From 11 to 50	72	32.7
From 51 to 100	16	7.3
More than 100	16	7.3

^a^ It refers to studies without sample (documentary studies).

^b^ It refers to studies that do not report the size of the sample analysed, although they used human sample.

In relation to the characteristics of the analysed sample (Table 4), the studies with elite-level artistic swimmers represent the highest percentage (30.9%), followed by those with a wide variety of levels. Studies with national-level samples exist in a much smaller proportion (7.7%). Likewise, studies with athletes of a lower level or initiation levels are very scarce. Finally, the high percentage of publications in which the level of the athletes is not specified is noteworthy.

**Table 4:** Level of the sample analysed in artistic swimming.

Level	Count	%
Does not specify	41	18.6
Initiation	6	2.7
Regional	5	2.3
National	17	7.7
Elite/International	68	30.9
Mixed	40	18.2

## Discussion

The objective of this work was to know the research trends in the sport modality of artistic swimming and to analyse the scientific production regarding this sport. The number of publications on artistic swimming in comparison to other sports is still very low (15, 16), which does not allow for large compilations of information, except for with regard to injuries and physiology, which help to establish a homogeneous and valid scientific base in artistic swimming. However, the publication of articles on the subject has been increasing considerably in recent years (especially from 2005 to 2022), reaching a maximum level of publications in 2021 (with a total of 43 publications). For this reason, it is considered a booming sport, and it is necessary to carry out reviews that collect said information as in this bibliometric study.

It is relevant that the largest numbers of publications on artistic swimming are case studies or documentary research. This may be due to the difficulty of obtaining a high and homogeneous sample. Even though the number of licenses and participation in international championships has been increasing in the last decade (17), it is one of the sports with the fewest licenses worldwide. For this reason, it could be speculated that one of the reasons researchers opt for the development of research with small samples is due to the limited existence of federative licenses in artistic swimming compared to other sports modalities, which leads to a possible lack of participants for conducting a study.

Given the multidisciplinary nature of artistic swimming (5), the fields in which scientific production has been analysed are very different. Even so, it seems that today the main concerns with respect to achieving high performance in artistic swimming are injury epidemiology and physiology. This may be directly related to the great physiological demands that athletes are subjected to in a different environment than usual and the possible injury situations to which athletes are subjected due to overuse during sports practice (5, 18). Artistic swimming requires a large number of hours of daily training that expose the body to great stress and the joints to great wear (5), thus documenting many injuries at the competitive level, with a high prevalence of shoulder, knee and lower back injuries among swimmers due to overuse as well as short-term injuries in tendon muscles (19–22).

Also noteworthy are the studies that analyse the recovery effect or body changes after several years, due to dedication, demands and practical execution (18, 23–25). In addition, topics such as nutrition, biomechanics or psychology should not be left aside, and their research should be promoted since their direct relationship with performance in artistic swimming has been amply demonstrated (26–28).

Regarding the sample, the data found and reflected in the results are in line with the low number of federated swimmers in artistic swimming (29), which makes access to said sample difficult. Thus, the main investigations have stood out for the study of cases or documentary research; for this reason, it can be affirmed that the creation of new facilities that adapt to the needs of the sport is necessary and/or that more publicity about the practice of this discipline should be generated; in this way, there is the possibility of carrying out investigations with more than 100 participants (at the moment only 7.27% correspond to them) as occurs in much more popular sports.

Regarding the performance level of the sample, the percentage of elite swimmers studied in the literature was higher than in other swimming categories. This may be because there is a greater interest within the scientific community in high-performance artistic swimming. Generally, since it is not a common topic investigated in the scientific community, the research groups that are interested in artistic swimming are made up of members with former practitioners or are usually related to or involved in the sport itself, and for this reason, they have access to high-level samples. Therefore, it would be necessary for researchers to take into account not only highly competitive swimmers but also other swimming categories for future studies. This allows the establishment of sports strategies adapted to each age group.

Finally, a possible weakness of the study that must be highlighted is the probability of the existence of a publication bias due to the low significance of the art in certain scientific fields that causes them to be published in journals of low scientific quality, which are not included in the databases used. Likewise, most of the studies continue to be heterogeneous, which makes it difficult to combine research by branch. The main solution to these problems is the promotion and creation of more studies on the subject as well as its corresponding promotion that would increase the relevance of the subject and decrease the heterogeneity of the matter.

## Conclusions

Over the years there has been a considerable increase in studies on artistic swimming, especially those with smaller samples (1–50 swimmers) and professional athletes. Besides, the topics of greatest interest in artistic swimming have been physiology, sports performance and injuries. Even so, it seems that for the moment artistic swimming has little impact, probably due to its status as a minority sport and it having limited social and economic impact. This study highlights the need to increase scientific research in artistic swimming that allows for reviews that establish more homogeneous and relevant conclusions for this sport field. Likewise, a greater impulse is needed from the environment of this sport (federations, organisations, clubs, and athletes) to improve knowledge about their area; following this path will achieve an increase in its performance by coaches and athletes as well as an increase in its social repercussions.

## Data Availability

The original contributions presented in the study are included in the article/Supplementary Material, further inquiries can be directed to the corresponding authors.
